# A Doctor’s Viewpoint

**Published:** 1915-02

**Authors:** Malone Duggan


A DOCTOR'S VIEWPOINT. By John Bessner Huber, A. M., M. D., Editor *The Dietetic and Hygiene Gazette;* Author Consumption and Civilization, etc.

This little volume of 164 pages is exceedingly entertaining and instructive; it is the summing up experiences and impressions of a scholar, and may be likened unto a delightful desert after a full meal. It is the finishing touches of the artist applied to his fully developed creation, and emphasizes the lights and shades which heightens its impressiveness. It is a veritable storehouse of information of delightful suggestions on the whole gamet of modern medical thought, couched in splendid literary style; a real inspiration to the higher medical ethics. MALONE DUGGAN.

